# Control Subarea Division for Coordinated Signal Control: A Colored Random Walk and Path Entropy Approach to Traffic-State Propagation

**DOI:** 10.3390/e28060692

**Published:** 2026-06-16

**Authors:** Pengcheng Li, Bin Li, Lin Wang, Wei Zhang, Sixian Li, Jun Hua

**Affiliations:** 1 ITS Center, Research Institute of Highway Ministry of Transport, Beijing 100088, China; 2 State Key Laboratory of Intelligent Transportation System, Research Institute of Highway Ministry of Transport, Beijing 100088, China; 3 Highway Monitoring & Response Center, Ministry of Transport of the People’s Republic of China, Beijing 100029, China; 4 Automobile Transportation Research Center, Research Institute of Highway Ministry of Transport, Beijing 100088, China

**Keywords:** control subarea division, coordinated signal control, colored random walk, path entropy analysis, traffic-state propagation

## Abstract

Control subarea division is essential for coordinated signal control, but methods based mainly on local correlation or static topology may not adequately capture traffic-state propagation under dynamic traffic loading. This study proposes a control subarea division method that explicitly models traffic-state propagation by integrating state-guided colored random walk and path entropy analysis. Intersection correlation degree and traffic state are used to construct a state-guided colored random walk process, in which transition probabilities are updated according to network connectivity and traffic-state consistency. Path entropy characterizes propagation uncertainty, and control subareas are identified by minimizing the distribution discrepancy between node-level and subarea-level path responses. To compare partitioning schemes, five complementary metrics were adopted: variance reduction rate of spatial delay, delay reduction rate, congestion mitigation index, stop reduction rate, and queue reduction rate. A VISSIM microsimulation model with dynamic traffic loading was developed to compare the proposed method with the Whitson and Fast Newman methods. The proposed method achieved the best performance across all five metrics, with values of 41.47%, 23.77%, 25.96%, 23.59%, and 15.08%, respectively. These results indicate that the proposed method improves spatial balance and network efficiency while mitigating bottlenecks, reducing stops, and suppressing queue accumulation.

## 1. Introduction

In large-scale urban road networks, coordinated signal control is a key means of improving traffic efficiency, mitigating congestion propagation, and maintaining the operational stability of the overall system [1]. With the expansion of urban road networks, traffic demand has become increasingly uneven in space and time, and traffic fluctuations have become more pronounced. Consequently, conventional control strategies designed for isolated intersections or fixed corridors can no longer effectively balance local responsiveness and network-wide coordination. Under high-demand or dynamically disturbed conditions, different areas of the network often exhibit significant heterogeneity in traffic state. If a uniform control scale is still adopted, local improvements may be achieved at the expense of overall network performance [2]. Therefore, identifying control subareas with strong internal coupling and relatively weak external interactions has become a fundamental prerequisite for regional coordinated control and large-scale signal optimization [3].

Existing studies on control subarea division can be broadly grouped into two methodological streams. The first route constructs control subareas according to traffic-state similarity or intersection correlation degree. In this line of research, traffic flow, travel time, queue length, delay, and signal timing characteristics are commonly used to identify intersections that should be grouped into the same coordinated control unit [4,5]. Such methods usually have clear physical meaning and strong engineering interpretability, and they are effective in capturing local coordination requirements between adjacent intersections. However, their partition results often depend heavily on local statistical relationships or empirical thresholds. When traffic demand changes rapidly and congestion propagates along multiple potential paths, local instantaneous correlations alone are often insufficient to reflect the actual spreading process of traffic influence in the network [6].

The second route abstracts the urban road network as a graph and organizes network units from a more global perspective by using graph partitioning, community detection, or deep graph clustering methods [7]. Compared with local correlation-based methods, these approaches generally provide better structural integrity and computational efficiency in large-scale networks, and they can generate more regular control subareas from the perspective of topology or global similarity. Nevertheless, their optimization objectives are still mainly centered on which nodes should be grouped together, rather than on how traffic states propagate, expand, and evolve across the network. As a result, under dynamic traffic loading, some intersections that are topologically adjacent but already diverging in traffic state may still be assigned to the same control subarea, thereby weakening the internal consistency of coordinated control objectives [8,9].

Beyond these two direct routes, propagation-oriented analysis and entropy-based uncertainty characterization provide important theoretical perspectives for understanding dynamic control subarea division [10]. From the perspective of traffic operation mechanisms, congestion evolution in road networks is strongly path-dependent and propagative. Traffic disturbances are not passively transferred only between adjacent intersections; rather, they may diffuse, accumulate, and feed back through multiple reachable paths. Random walk offers an effective tool for describing such network spreading processes, and previous studies have shown that random walk-based models can capture flow dynamics, diffusion behavior, and structural organization in complex networks [11,12]. Meanwhile, information entropy and related measures have been widely used to analyze uncertainty, predictability, and complexity in transportation systems, providing a natural way to quantify the dispersion and stability of propagation processes [13]. These observations suggest that, for control subarea division, it is not sufficient to consider only local correlation strength or static topological structure; it is also necessary to examine how traffic states propagate through the network and whether the resulting propagation patterns are stable and internally coherent enough to support coordinated control [14].

To address these limitations, this paper proposes a control subarea division method that explicitly models traffic-state propagation by integrating state-guided colored random walk and path entropy analysis. Using intersection correlation degree and traffic state as inputs, the proposed method first constructs a state-guided colored random walk process on the road network graph to simulate the multipath propagation of traffic influence. It then extracts path response characteristics of key intersections and uses path entropy to characterize the uncertainty of the propagation process. Finally, control subareas are identified by combining node-level path response distributions with the distributional characteristics of candidate subareas. Compared with conventional methods based on local correlation thresholds or topological modularity optimization, the proposed method explicitly models the propagation mechanism of traffic influence and the accumulation of propagation uncertainty under dynamic traffic conditions, and is therefore designed to generate control subareas that are more consistent with coordinated-control requirements.

The main contributions of this study are summarized as follows:

(1) From the perspective of traffic-state propagation, this study reformulates the control subarea division problem and develops a state-guided colored random walk framework with intersection state labels, so that subarea identification is no longer restricted to static adjacency or average correlation strength, but can reflect the multipath diffusion characteristics of traffic influence in complex road networks.
(2) Path entropy is introduced to characterize the uncertainty of traffic propagation, thereby incorporating propagation-level information dispersion into control subarea division and complementing conventional criteria dominated by topological compactness or local similarity with an uncertainty-aware interpretation.
(3) A VISSIM-based dynamic traffic loading scenario is constructed, and a representative correlation-threshold method and a community-detection-based method are used as baselines. The proposed method is evaluated from the perspectives of spatial division results, propagation mechanism interpretation, and multidimensional operational performance, and the results show that it achieves better bottleneck mitigation, network balance, and operational stability under high-demand conditions.

The remainder of this paper is organized as follows. Section 2 reviews related studies on control subarea division, random walk, and entropy analysis. Section 3 presents the theoretical framework and algorithm design of the proposed method. Section 4 reports the experimental design, spatial division results, and quantitative performance comparison. Section 5 concludes the paper and outlines future research directions.

## 2. Related Work

### 2.1. Control Subarea Division Based on Traffic-State Similarity and Correlation

Control subarea division is a fundamental step in large-scale coordinated signal control because it determines how intersections are grouped into manageable control units before signal coordination is implemented. One major research route divides control subareas according to traffic-state similarity or intersection correlation degree. In this line of research, traffic flow, travel time, delay, queue length, and signal timing characteristics are commonly used to describe the operational relationship between intersections and to identify candidate intersections for coordinated control.

These studies generally extend the basis of subarea division from simple geometric adjacency to operational similarity, using network-level traffic states, OD-related information, corridor flow organization, and intersection correlation measures to identify intersections with coordinated-control potential. Mo et al. [15] divided signal control subregions based on the macroscopic fundamental diagram and showed that network-level traffic-state indicators can support regionalized control under varying traffic demand. Xu and Tian [16] proposed an OD-based partition technique using connected vehicle data to improve arterial signal coordination, demonstrating the potential of trajectory-related information for adaptive subarea partitioning. Ke et al. [17] introduced a traffic origin–destination-flow-inspired dynamic arterial partition method for coordinated signal control, extending the partitioning logic from pairwise interaction to corridor-level traffic organization. In addition, Li et al. [18] modeled adjacent intersection correlation using a temporal graph attention network, while Lan and Wu [19] studied signal control subarea partitioning from the perspective of correlation degree analysis.

These studies demonstrate that methods based on traffic-state similarity and correlation have clear physical meaning and strong engineering interpretability. However, most of them still rely on pairwise similarity, local statistical dependence, or threshold-based decision rules [17,19]. As a result, they are effective in identifying local coordination requirements between adjacent intersections, but are less capable of characterizing how traffic disturbances propagate across multiple paths in a complex network. When congestion spreads beyond immediate neighbors, local correlation alone may be insufficient to determine the true boundary of a control subarea.

### 2.2. Control Subarea Division Based on Network Structure and Community Detection

To overcome the locality of correlation-based methods, another important research stream formulates control subarea division as a graph-based clustering problem. In this framework, intersections are represented as nodes and their interactions are encoded as weighted links, after which graph partitioning, community detection, and clustering algorithms are used to divide the network into groups with strong internal connections and weak external coupling.

Existing graph-based studies commonly aim to improve the structural organization and scalability of subarea division by representing traffic networks as weighted graphs and identifying groups of nodes with strong internal connectivity or high modular cohesion. Khawaja et al. [20] reviewed a wide range of community detection methods in complex networks, providing an important methodological basis for network-oriented traffic partitioning research. Gu et al. [21] incorporated constrained network partition into large-scale traffic signal control with adaptive deep reinforcement learning, showing that network partition can improve the scalability of coordinated control. Ma and Wu [22] further proposed an adaptive network partition strategy for coordinated traffic signal learning, indicating that dynamic partition can be embedded into data-driven signal control frameworks. From the perspective of traffic network representation and clustering, Zhang et al. [23] proposed GATC and DeepCut, combining deep spatiotemporal feature extraction with clustering for large-scale transportation network partition. Zhang and Pang [24] developed a dynamic division method for highway control subareas based on an improved label propagation algorithm, further demonstrating the applicability of community-detection-based partitioning to traffic management scenarios.

Compared with correlation-based approaches, these methods provide a stronger global view of network organization and are generally more suitable for large-scale systems. However, their optimization objective is still primarily structural cohesion rather than traffic-state transmission. In other words, they focus on which intersections should be grouped together, but do not explicitly model how traffic disturbances spread, accumulate, and evolve through the network under dynamic loading conditions. Consequently, the resulting control subareas may be topologically compact but not necessarily consistent in terms of propagation behavior.

### 2.3. Random Walk Models for Propagation Analysis

From the perspective of traffic operation mechanisms, congestion evolution in road networks is inherently propagative and path-dependent. Random walk provides an effective tool for describing diffusion, accessibility, and flow-based structural organization in complex networks. Some studies suggest that random walk-based models are particularly useful for interpreting network dynamics from a propagation perspective, because they can reveal diffusion paths, flow persistence, and influence ranges that are not directly observable from static topology alone. Riascos and Mateos [25] systematically reviewed local and non-local random walks on weighted networks and showed that random walk-based analysis can reveal dynamic connectivity patterns that are difficult to identify from static topology alone. Rosvall et al. [26] showed that memory in network flows can substantially affect spreading dynamics and community detection, highlighting the importance of path dependence in diffusion processes. Bovet et al. [27] proposed a flow-stability framework for dynamic community detection, further indicating that community structures can be interpreted from the persistence and evolution of network flows.

These ideas are also relevant to transportation systems. Anwar et al. [28] proposed RoadRank to estimate traffic diffusion and influence in dynamic urban road networks, showing that propagation processes can reveal interaction patterns beyond direct adjacency. Taken together, these studies suggest that control subarea division should not be treated solely as a static grouping problem, but also as a process of identifying regions with similar propagation behavior.

Nevertheless, existing random walk-related studies rarely address control subarea division directly. In coordinated traffic control, disturbance propagation is not determined by topology alone; it is also strongly modulated by traffic state, loading level, and downstream conditions. Therefore, a state-guided propagation model is more suitable than a topology-only random walk for identifying control subareas in dynamic traffic environments.

### 2.4. Entropy-Based Analysis of Traffic Uncertainty and Path Stability

Entropy-based studies provide a quantitative way to describe the dispersion, uncertainty, and predictability of traffic states or path flows, thereby offering a useful basis for evaluating whether propagation patterns are concentrated or unstable. Li et al. [29] estimated the limit of predictability in short-term traffic forecasting through an entropy-based approach, showing that traffic evolution contains an intrinsic uncertainty structure that cannot be fully captured by conventional performance indicators alone. Guo et al. [30] analyzed the predictability of path flow distribution in urban road networks using information entropy, providing a path-level perspective on uncertainty in network traffic organization. At the traffic-state level, Liu et al. [31] directly examined the information entropy of traffic flows and demonstrated that entropy-based measures can effectively describe traffic uncertainty and complexity.

Although these studies provide important inspiration, entropy has rarely been incorporated into the criterion of control subarea division itself. Most existing work uses entropy to describe traffic states, predictability, or network complexity, rather than to evaluate whether the propagation paths associated with different intersections are sufficiently concentrated and consistent to support coordinated control. This gap suggests that path-level uncertainty remains insufficiently explored in existing control subarea division research.

### 2.5. Summary and Positioning of This Study

In summary, existing studies provide separate insights into local operational similarity, global structural organization, propagation dynamics, and uncertainty characterization, but these dimensions have not been sufficiently integrated into a unified control subarea division framework. Traffic-state-similarity- and correlation-based methods are effective in identifying local coordination relationships and remain attractive because of their direct engineering interpretability. Network structure-oriented methods improve the global organization ability of control subarea division and are well-suited to large-scale systems. Random walk-related studies reveal that network structure can also be understood from a propagation perspective rather than only from static connectivity. Entropy-based studies offer useful tools for quantifying uncertainty and complexity in traffic and network processes.

However, current research still lacks a unified framework that jointly considers traffic-state propagation, path-level uncertainty, and control subarea boundary identification. Most existing methods emphasize either local correlation, global topology, stochastic diffusion, or uncertainty characterization, but seldom integrate these aspects within the same control subarea division process. To address these limitations, this study combines state-guided colored random walk with path entropy analysis to identify control subareas that are structurally connected and internally consistent in terms of traffic-state propagation under dynamic traffic conditions.

## 3. Materials and Methods

**Figure 1:**
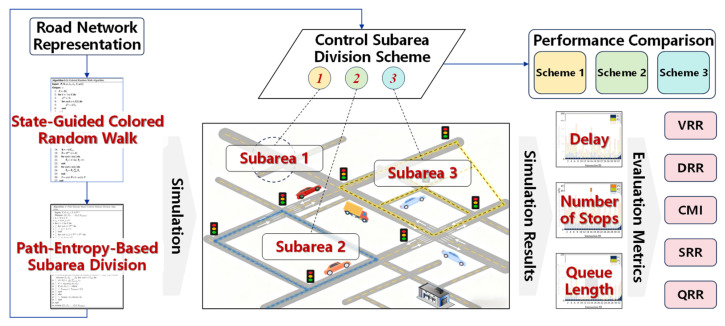
Overall workflow of the proposed control subarea division and simulation-based evaluation framework.

The proposed method takes the urban road network, intersection correlation degree, and traffic state as inputs, and identifies control subareas from the perspective of traffic-state propagation rather than purely static topology. Figure 1 shows the overall framework of the proposed method.

The overall workflow consists of three functional parts: input representation, propagation-based subarea division, and simulation-based performance evaluation. First, the urban road network is represented as a weighted graph, in which intersection correlation degree describes the structural and operational relationships among intersections, and traffic-state labels provide the dynamic state information required for propagation modeling. Second, a state-guided colored random walk process is constructed to simulate the propagation of traffic influence across the network. In this process, transition probabilities are adjusted according to both network connectivity and traffic-state consistency, so that the resulting propagation paths reflect not only topological adjacency but also state-dependent spreading tendencies. Third, path entropy and path response distributions are used to quantify propagation uncertainty and identify control subareas with internally consistent propagation patterns. The obtained partitioning scheme is then evaluated in a VISSIM microsimulation environment and compared with benchmark methods using average delay, average number of stops, average queue length, and five aggregate performance metrics.

### 3.1. State-Guided Colored Random Walk for Traffic-State Propagation

The essence of the proposed CRW module is to embed traffic-state information into the random walk process, so that the propagation of traffic influence is jointly determined by network topology and node state. Let the urban road network be represented as a graph $G=\left(V,E\right)$, where V denotes the set of intersections and E denotes the set of traffic connections between intersections. The weighted adjacency matrix is denoted by $A=A_{ij}$, where $A_{ij}$ represents the correlation weight between intersections $v_{i}$ and $v_{j}$. The one-step transition probability from node $v_{i}$ to $v_{j}$ is defined as

(1) $$P_{ij}=A_{ij}/\sum_{j}A_{ij}$$

In this study, the intersection correlation degree and traffic state are taken from the previous research and are used here as the input of the control subarea division framework [18]. To characterize traffic states, each key intersection is assigned a color label corresponding to its traffic condition. Here, “color” is not a graphical attribute, but a symbolic representation of traffic state. Let $c^{\left(k,t\right)}$ denote the current color distribution associated with seed node k at iteration t and let $s^{\left(k\right)}$ denote the corresponding initial color label vector. The color distribution is updated as shown in Equation (2). In this way, the random walk process is initialized from key intersections with known traffic-state labels, while the state responses of other intersections are gradually inferred through propagation. The parameter α controls the trade-off between outward exploration and return to the seed node. A larger α encourages the walker to explore a broader region of the network, whereas a smaller α places more emphasis on preserving the local state dependence around the seed intersection.

(2) $$c_{k}^{\left(t+1\right)}=\alpha Pc_{k}^{\left(t\right)}+\left(1-\alpha \right)s_{k}$$

where $c_{k}^{\left(t\right)}={\left[c_{k,1}^{\left(t\right)},c_{k,2}^{\left(t\right)},\ldots ,c_{k,L}^{\left(t\right)}\right]}^{T}$ denotes the traffic-state response distribution associated with seed node k at iteration t. L is the number of traffic-state labels, $s_{k}$ is the initial one-hot state-label vector of seed node k, and $\alpha \in \left(0,1\right)$ is the restart coefficient controlling the trade-off between outward diffusion and return to the seed node.

To make the random walk process sensitive to traffic-state differences, the original transition matrix is further enhanced by a state-guided transition mechanism, as defined in Equations (3)–(5). Specifically, for seed node k, the original transition matrix is modulated according to its current traffic-state response distribution $c_{k}^{\left(t\right)}$, where $c_{k,l}^{\left(t\right)}$ denotes the probability mass associated with traffic-state label l at iteration t. The state-guided coefficient matrix $R_{k}^{\left(t\right)}$ is defined by Equation (4). The symbol “∘” denotes the Hadamard product, which is used to enhance the existing edge weights without introducing any new edges into the network. According to Equation (5), when the traffic-state level of neighboring node j is not higher than the propagated state label l, i.e., $s_{j}\leq l$, an attraction coefficient $\lambda_{1}$ is assigned; otherwise, a repulsion coefficient $-\lambda_{2}$ is assigned.

(3) $$P_{k}^{\left(t\right)}=P\circ \left(1+R_{k}^{\left(t\right)}\right)$$

(4) $$R_{k,ij}^{\left(t\right)}=\sum_{l=1}^{L}c_{k,l}^{\left(t\right)}\phi \left(l,s_{j}\right)$$

(5) $$\phi \left(l,s_{j}\right)=\left\{\begin{array}{ll} \lambda_{1} & s_{j}\leq l\\ -\lambda_{2} & s_{j}>l \end{array}\right.$$

Because the transition matrix evolves during iteration, direct recursive updating may lead to oscillation or unstable convergence. To address this issue, a decay-based update mechanism is introduced, as shown in Equation (6).

(6) $${\mathbb{P}}_{k}^{\left(t\right)}=\left\{\begin{array}{ll} P & t=0\\ \psi \left(t\right)P_{k}^{\left(t\right)}+\left(1-\psi \left(t\right)\right){\mathbb{P}}_{k}^{\left(t-1\right)} & t\geq 1 \end{array}\right.$$

In this study, the decay coefficient is defined as $\psi \left(t\right)=e^{-\beta t}$, where β>0 controls the decay rate. As $\psi \left(t\right)$ approaches 0, the contribution of the current state-enhanced transition matrix $P_{k}^{\left(t\right)}$ diminishes, and the smoothed transition matrix becomes increasingly close to its value at the previous iteration, thereby promoting stable convergence. Based on the smoothed transition matrix in Equation (6), the traffic-state response distribution for seed node k is updated as

(7) $$c_{k}^{\left(t+1\right)}=\alpha {\mathbb{P}}_{k}^{\left(t\right)}c_{k}^{\left(t\right)}+\left(1-\alpha \right)s_{k}$$

This treatment smooths the temporal evolution of the transition matrix and ensures that the traffic-state propagation process gradually stabilizes while retaining sufficient sensitivity to state variation. Through the above procedure, the CRW module transforms the road network from a static weighted graph into a state-guided propagation system. The output of this module is a set of path response patterns describing how traffic influence initiated from key intersections spreads across the network under the joint effect of topology and traffic state.

### 3.2. Path Entropy and Distribution Discrepancy for Control Subarea Division

Although the improved colored random walk can describe how traffic influence propagates through the network, path generation alone is not sufficient for control subarea division. It is further necessary to quantify the uncertainty of node-level propagation and to evaluate the consistency between node-level and subarea-level propagation patterns. To this end, path entropy is introduced to characterize propagation uncertainty, whereas distribution discrepancy is used to determine the final control subareas.

After the random walk process is completed, repeated walks from each key intersection generate a path response vector for node i denoted by $r_{i}=\left[r_{i1},r_{i2},\ldots ,r_{im}\right]$, where $r_{ij}$ represents the normalized visit probability of node i on path j, and m is the total number of propagation paths. Specifically

(8) $$r_{ij}=\frac{n_{ij}}{\sum_{l=1}^{m}n_{il}}$$

where $n_{ij}$ denotes the number of visits of node i on propagation path j, m is the total number of sampled propagation paths, and $r_{ij}$ is the normalized visitation probability of node i on path j.

Based on $r_{i}$, the path entropy of node i is defined as

(9) $$H_{i}=-\sum_{j=1}^{m}r_{ij}log\left(r_{ij}\right)$$

In this study, $log\left(.\right)$ denotes the natural logarithm.

A larger value of $H_{i}$ indicates that the propagation response of node i is distributed over a broader and more dispersed set of paths, implying a more unstable and uncertain propagation pattern. By contrast, a smaller value of $H_{i}$ indicates that the propagation response is more concentrated and stable. Unlike conventional indicators such as delay, queue length, or flow, path entropy does not describe the severity of the traffic state itself, but rather the uncertainty of how that state propagates through the network.

To extend node-level propagation characteristics to the control-subarea level, the average path response vector and the average path entropy of candidate control subarea $Z_{k}$ are defined as

(10) $${\bar{r}}_{k}=\frac{1}{\left|Z_{k}\right|}\sum_{i\in Z_{k}}r_{i}$$

(11) $${\bar{H}}_{k}=\frac{1}{\left|Z_{k}\right|}\sum_{i\in Z_{k}}H_{i}$$

where $Z_{k}$ denotes the k-th candidate control subarea, $\left|Z_{k}\right|$ denotes the number of nodes in that subarea. ${\bar{r}}_{k}$ is the average path response vector of $Z_{k}$, and ${\bar{H}}_{k}$ is the average path entropy of $Z_{k}$. Here, ${\bar{r}}_{k}$ provides the subarea-level reference distribution for subsequent KL-divergence-based partitioning, whereas ${\bar{H}}_{k}$ is used as a descriptive statistic to characterize the entropy level of the candidate control subarea. These quantities provide a statistical description of the overall propagation behavior within a candidate control subarea. If the nodes in $Z_{k}$ exhibit similar path response distributions and comparable entropy levels, the control subarea can be regarded as having internally consistent propagation dynamics, which is desirable for coordinated control. Conversely, if node-level propagation responses are highly heterogeneous, the candidate control subarea is likely to contain divergent propagation patterns and should not be treated as a coherent control unit.

Based on this idea, the discrepancy between node i and candidate control subarea $Z_{k}$ is measured by the Kullback–Leibler (KL) divergence:

(12) $$D_{KL}\left(i,Z_{k}\right)=\sum_{j=1}^{m}r_{ij}log\left(\frac{r_{ij}}{{\bar{r}}_{kj}}\right)$$

where ${\bar{r}}_{kj}$ is the j-th component of the average path response vector ${\bar{r}}_{k}$. A smaller value of $D_{KL}\left(i,Z_{k}\right)$ indicates that the propagation pattern of node i is more compatible with the average propagation pattern of control subarea $Z_{k}$.

Specifically, to avoid undefined logarithmic terms caused by zero components in ${\bar{r}}_{kj}$, additive smoothing was applied to the subarea-level average path response vector before the KL-divergence calculation. The smoothed component is defined as:

(13) $${\bar{r}}_{kj}^{\left(\varepsilon \right)}=\frac{{\bar{r}}_{kj}+\varepsilon }{\sum_{l=1}^{m}\left({\bar{r}}_{kj}+\varepsilon \right)}$$

where $\varepsilon =10^{-6}$ was used in this study. In the implementation of Equation (12), ${\bar{r}}_{kj}$ was replaced by ${\bar{r}}_{kj}^{\left(\varepsilon \right)}$ This treatment only ensures numerical stability and does not change the meaning of the distribution discrepancy measure.

Accordingly, the final control subarea division is obtained by minimizing the total intra-subarea distribution discrepancy over the network

(14) $$Z^{*\left(r\right)}=\arg\underset{Z}{\min}\sum_{k=1}^{K}\sum_{i\in Z_{k}}D_{KL}\left(i,Z_{k}^{\left(r\right)}\right)$$

where $Z_{k}^{\left(r\right)}$ denote the control subarea label assigned to node i at iteration r, and $Z^{\left(r\right)}=\left[Z_{1}^{\left(r\right)},Z_{2}^{\left(r\right)},\ldots ,Z_{N}^{\left(r\right)}\right]$ denote the assignment vector of all nodes at iteration r. The KL-divergence minimization process is terminated when the assignment vector remains unchanged between two consecutive iterations, namely when $Z_{k}^{\left(r\right)}=Z_{k}^{\left(r-1\right)}$.

In addition, a maximum iteration number $R_{\max}=100$ was used as a protective upper bound to avoid repeated computation in extreme cases. This upper bound does not mean that the algorithm always runs for 100 iterations. For the 39-node study network used in this study, the computational burden of the KL-divergence-based partitioning procedure was relatively small, and setting $R_{\max}=100$ did not significantly affect the overall computational efficiency.

In the proposed framework, path entropy is used to characterize the uncertainty and dispersion of node-level propagation, whereas the KL-divergence objective is used to enforce the internal consistency of path response distributions within each control subarea.

The main steps of the proposed control subarea division method are summarized in Algorithm 1.
**Algorithm 1.** Workflow of the proposed control subarea division algorithm
**Input:** $G=\left(V,E\right),$ A, $s_{k},$ α, $\lambda_{1},$ $\lambda_{2},$ $\psi \left(t\right),$ T, K
 **Output:** $Z^{*}$1Construct the initial transition matrix.2Initialize $c^{\left(k,0\right)}\leftarrow s^{\left(k\right)}$ for each seed node k
3**for** t←1 to T **do**4  **for** *each seed node* k **do**5    Construct and smooth the state-guided transition matrix according to Equations (3)–(6).6    Update the propagation response distribution according to Equation (7).7  **end**
8  **if** *the state propagation process converges* **then**9    **break**
10  **end**
11**end**12**for** *each node* i∈V **do**13  Obtain the path response vector according to Equation (8).14  Compute the path entropy according to Equation (9).15**end**16Initialize candidate control subareas $Z=\left\{Z_{1},Z_{2},\ldots ,Z_{K}\right\}.$
17**repeat**18  **for** k←1 to k **do**19    Compute the control-subarea-level statistics according to Equations (10)–(11).20  **end**
21  **for** *each node* i∈V **do**22    Compute the discrepancy between node i and each candidate control subarea according to Equation (12).23    Assign node i to the control subarea with the minimum discrepancy.24  **end**
 25**until** the assignment is unchanged or the objective converges 26$Z^{*}\leftarrow Z.$

In summary, the proposed method first models traffic-state propagation in the urban road network through the state-guided colored random walk process, then evaluates the uncertainty of the resulting propagation patterns using path entropy, and finally determines the control subareas by minimizing the distribution discrepancy between node-level and subarea-level propagation responses.

## 4. Results and Discussion

This section evaluates the proposed method on a VISSIM-based microsimulation platform built from field survey data. After introducing the study area, simulation settings, and evaluation metrics, the baseline deterioration under isolated signal control is analyzed. The partition characteristics of the benchmark methods and the mechanism of the proposed method are then examined, followed by a quantitative comparison of network-level control performance.

### 4.1. Study Area and Simulation Setup

**Figure 2:**
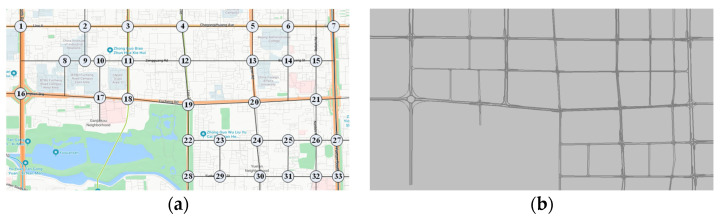
Study network used in the VISSIM simulation. (**a**) Intersection numbering scheme; (**b**) Reconstructed VISSIM network.

The study network contains 39 intersections, including 33 signalized intersections. Specifically, the network includes 22 four-leg intersections, 10 T-intersections, and 1 roundabout intersection. The road geometry, link lengths, intersection spacing, number of lanes, approach channelization, turning connectors, and intersection types were checked and reconstructed using field survey information, map imagery, and distance measurements from Baidu Maps. Signal timing plans and directional traffic volumes were implemented according to the field survey data, and turning movements within the network were represented using Static Vehicle Routing Decisions in VISSIM. The simulation outputs under the base-demand condition were also compared with the observed traffic volumes at boundary entrances and key road sections to check whether the model could reasonably reproduce the observed traffic-flow scale and directional-flow structure. The intersection numbering scheme and the reconstructed VISSIM network are shown in Figure 2.

The total simulation duration was set to 6300 s and was divided into four consecutive periods. Period 0 (P0, 0–1800 s) was used as the warm-up period and was excluded from all statistics. Period 1 (P1, 1800–3300 s) used the observed traffic demand. Period 2 (P2, 3300–4800 s) increased the boundary entrance demand to 1.2 times the observed level, and Period 3 (P3, 4800–6300 s) further increased it to 1.5 times the observed level. This design was used to reproduce the transition from stable operation to high-load and locally oversaturated conditions.

Each of P1–P3 lasted 1500 s and was divided into three sequential windows: 600 s for demand loading and traffic-state diagnosis, 300 s for signal-plan transition after the coordinated control scheme was updated, and 600 s for performance evaluation. The boundary entrance demands and signal timing parameters were dynamically adjusted through the VISSIM COM interface using Python 3.9. Specifically, the COM interface was used to update boundary Vehicle Inputs and signal control parameters at the beginning of each loading period, so that Period 1 used the observed demand, Period 2 used 1.2 times the observed boundary demand, and Period 3 used 1.5 times the observed boundary demand.

For comparison, two baseline methods were adopted: the Whitson method, which emphasizes local coordination based on adjacent intersection correlations, and the Fast Newman method, which emphasizes topological clustering from the perspective of graph community detection. The main parameter settings of the three methods are summarized in Table 1.

To examine whether the proposed control subarea division result was sensitive to the selected parameters, an algorithm-level one-factor-at-a-time sensitivity analysis was conducted. The parameter combination reported in Table 2 was used as the default setting, and the resulting control subarea division was taken as the reference result. For each test, only one parameter was varied, while all other parameters were kept unchanged. For each perturbed setting, the state-guided colored random walk, path response distribution calculation, path entropy calculation, and KL-divergence-based partitioning procedure were rerun. The sensitivity was evaluated using partition agreement, changed intersections, average KL divergence, average path entropy, and the number of control subareas.

As shown in Table 2, the proposed method was generally robust to parameter variations within the tested ranges. Variations in $\lambda_{1}$, α, and β did not change the final control subarea structure, with partition agreement remaining at 1.000 and no changed intersections. The parameter $\lambda_{2}$ only slightly affected boundary-node assignment, with partition agreement ranging from 0.968 to 1.000. By contrast, the number of random walk steps T and the number of sampled propagation paths $N_{walk}$ had more visible effects on propagation coverage and stochastic stability. When T increased, the average path entropy gradually increased and became nearly stable after approximately 8–10 steps; therefore, T=10 was adopted to capture the main multipath propagation characteristics without unnecessary computation. When $N_{walk}$ reached 1000, the partition result and entropy-related indicators became stable; therefore, $N_{walk}=1000$ was selected to balance computational stability and computational cost.

### 4.2. Evaluation Metrics

To evaluate the effectiveness of different control subarea division schemes under dynamic traffic loading, this study considered both conventional traffic measures and five aggregated performance metrics. The conventional measures, namely average delay, average queue length, and average number of stops, reflect the overall operational condition of the network. However, these measures alone are insufficient to reveal whether a partitioning scheme improves network-wide efficiency, reduces spatial imbalance, mitigates critical bottlenecks, maintains traffic continuity, or suppresses queue accumulation. Therefore, five complementary indicators were adopted: the spatial delay variance reduction rate (VRR), the delay reduction rate (DRR), the congestion mitigation index (CMI), the stop reduction rate (SRR), and the queue reduction rate (QRR).

Let $D_{it}^{\left(m\right)}$, $S_{it}^{\left(m\right)}$, and $Q_{it}^{\left(m\right)}$ denote the average delay, average number of stops, and average queue length at intersection i during period t under method m, where $m\in \left\{Base,Whitson,FN,Proposed\right\}$, i=1,2,…,N, and t=1,2,…,T. Here, N is the number of intersections and T is the number of evaluation periods. Their units are s/veh, stops/h, and m, respectively.

(1) VRR

The spatial delay variance reduction rate (VRR) is used to measure the extent to which a control subarea division method improves the spatial balance of network operation. Specifically, it evaluates whether delay differences among intersections are reduced relative to the isolated signal control baseline. A higher VRR indicates that the method not only improves traffic performance, but also narrows the disparity in delay across the network.

For each intersection i under method m, the period-averaged delay is first defined as

(15) $$\mu_{i}^{m}=\frac{1}{T}\sum_{t=1}^{T}D_{it}^{m}$$

Based on the period-averaged delays of all intersections, the spatial variance of delay under method m is calculated as

(16) $$Var^{m}=\frac{1}{N}\sum_{i=1}^{N}{\left(\mu_{i}^{m}-{\bar{\mu }}_{i}^{m}\right)}^{2}$$

The VRR is then defined as

(17) $$VRR^{m}=\frac{Var^{base}-Var^{m}}{Var^{base}}$$

where $Var^{base}$ is the spatial variance of period-averaged intersection delay under the isolated signal control baseline. A larger $VRR^{m}$ indicates a greater reduction in spatial delay dispersion and therefore a more balanced network state. By contrast, a low or negative VRR suggests that the method provides limited improvement in network-wide operational balance.

(2) DRR

The delay reduction rate (DRR) is used to measure the extent to which a control subarea division method improves overall network efficiency. Specifically, it evaluates the relative reduction in network-level average delay compared with the isolated signal control baseline. A higher DRR indicates a greater improvement in overall delay performance.

Based on the period-averaged delay defined in Equation (15), the network-level average delay under method m is expressed as

(18) $${\bar{D}}^{m}=\frac{1}{N}\sum_{i=1}^{N}\mu_{i}^{m}$$

The DRR is then defined as

(19) $$DRR^{m}=\frac{{\bar{D}}^{base}-{\bar{D}}^{m}}{{\bar{D}}^{base}}$$

where ${\bar{D}}^{base}$ is the network-level average delay under the isolated signal control baseline. A larger $DRR^{m}$ indicates that method m is more effective in reducing overall network delay, whereas a lower DRR suggests a weaker improvement in operational efficiency.

(3) CMI

The congestion mitigation index (CMI) is used to measure the extent to which a control subarea division method achieves targeted bottleneck mitigation. Unlike DRR, which reflects the overall change in network-level delay, CMI focuses specifically on the most congested intersections under the highest loading condition. A higher CMI indicates that the method is more effective in relieving critical bottlenecks rather than merely improving average conditions across the network.

Let $t^{*}$ denote the highest loading period. The set of congested intersections is first identified from the isolated signal control baseline as

(20) $$\Omega =\left\{i\left|D_{it^{*}}^{base}>\theta \right.\right\}$$

where θ is the delay threshold used to identify bottleneck intersections. In this study, $t^{*}$ corresponds to Period 3 and θ=55s/veh was adopted as the delay threshold for identifying bottleneck intersections. This value follows the Highway Capacity Manual, where 55 s/veh represents the boundary between LOS D and LOS E for signalized intersections [32].

The CMI is then defined as

(21) $$CMI^{m}=\frac{\sum_{i\in \Omega }\left(D_{it^{*}}^{base}-D_{it^{*}}^{m}\right)}{\sum_{i\in \Omega }D_{it^{*}}^{base}}$$

where $D_{it^{*}}^{base}$ and $D_{it^{*}}^{m}$ denote the average delay of intersection i in the highest loading period under the baseline and method m, respectively. A larger $CMI^{m}$ indicates stronger mitigation of severe congestion at bottleneck intersections.

(4) SRR and QRR

The stop reduction rate (SRR) and queue reduction rate (QRR) were used to quantify the relative reductions in the network-level average number of stops and average queue length, respectively, compared with the isolated signal control baseline. The network-level averages were calculated over all intersections and all evaluation periods. A higher SRR indicates better traffic continuity and smoother progression, whereas a higher QRR indicates stronger suppression of queue accumulation and spillback risk under dynamic traffic loading.

Taken together, these five metrics evaluate control subarea division from the perspectives of spatial balance improvement, overall network efficiency improvement, targeted bottleneck mitigation, traffic continuity improvement, and queue suppression capability.

### 4.3. Baseline Traffic Deterioration Under Isolated Signal Control

**Figure 3:**
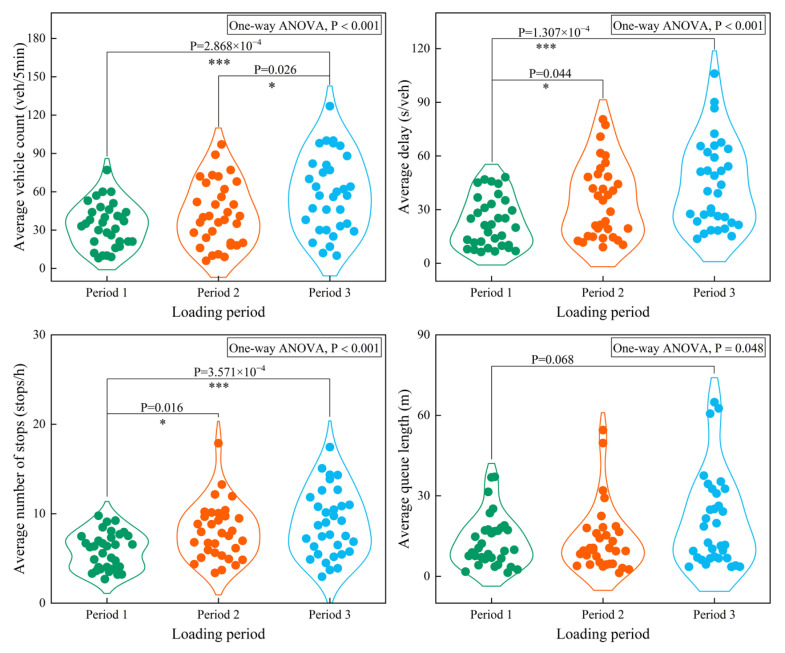
Distributions of baseline traffic-state and operational performance indicators under isolated signal control across the three loading periods. * The pairwise significance levels: * *p* < 0.05 and *** *p* < 0.001.

Before comparing different control subarea division methods, a baseline scenario with isolated signal control was established, in which all intersections operated without regional coordination. To ensure comparability, the baseline indicators were calculated from the evaluation windows defined in Section 4.1. Figure 3 shows the distributions of average vehicle count, average delay, average number of stops, and average queue length across the three loading periods.

As traffic demand increased from P1 to P3, the average vehicle count, average delay, and average number of stops showed clear upward shifts. Average queue length did not increase uniformly across all intersections, but its distribution exhibited a more pronounced upper tail in P3, suggesting localized queue accumulation at several intersections. These patterns indicate that isolated signal control was unable to maintain stable network operation under dynamic traffic loading.

Before conducting ANOVA, the normality and homogeneity-of-variance assumptions were examined using the Shapiro–Wilk and Levene tests, respectively. Since the homogeneity assumption was not fully satisfied for all indicators, Welch ANOVA and Kruskal–Wallis tests were additionally used as robustness checks.

As shown in Table 3, average delay, average number of stops, and vehicle count remained statistically significant under the robust tests, with effect sizes $\eta^{2}$ of 0.161, 0.150, and 0.151, respectively. By contrast, average queue length was only marginally significant in the classical ANOVA and became non-significant under the robust tests, indicating that queue growth should be interpreted mainly as localized accumulation rather than a uniform network-wide increase.

The statistical results further support the patterns observed in Figure 3, Asterisks indicate the significance levels of pairwise comparisons: * *p* < 0.05 and *** *p* < 0.001. Exact *p* values are shown in the figure. The average vehicle count, average delay, and average number of stops all showed significant differences across periods, indicating that network deterioration emerged progressively as traffic demand increased. For average delay, the difference between P1 and P2 was already significant (p=0.044), and the contrast between P1 and P3 became stronger (p<0.001). A similar pattern was observed for the average number of stops, with a significant increase from P1 to P2 (p=0.016) and a larger difference by P3 (p<0.001). For average queue length, the overall ANOVA reached the 0.05 significance level but remained close to the threshold (p=0.048). Therefore, the queue-length result should be interpreted mainly as localized queue accumulation rather than a uniform network-wide increase.

These results indicate that the deterioration under isolated signal control was not spatially uniform. Instead, several critical intersections exhibited high delay, frequent stopping, and long queue accumulation earlier than the rest of the network, thereby widening the operational disparity among intersections. Therefore, once traffic demand reaches a sufficiently high level, isolated signal control becomes inadequate for network-level coordinated operation, which motivates the need for appropriate control subarea division.

### 4.4. Benchmark Partition Structures Under the Representative High-Load Condition

**Figure 4:**
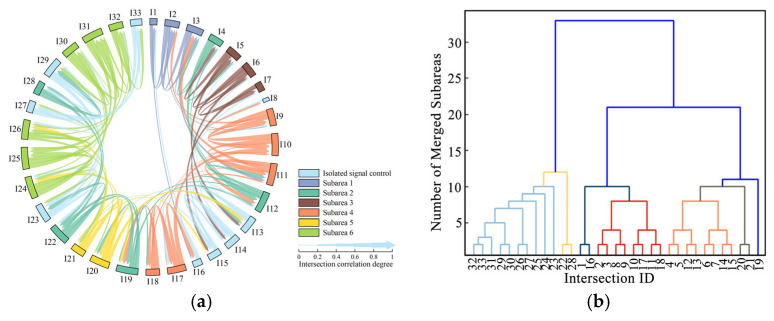
Partitioning process outputs of the benchmark methods under the representative high-load condition. (**a**) Whitson method; (**b**) Fast Newman method.

**Figure 5:**
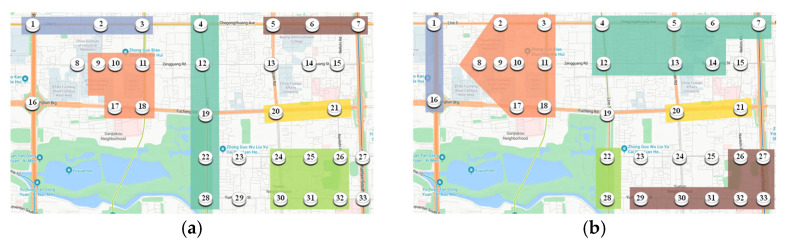
Spatial partitioning results of the benchmark methods under the representative high-load condition. (**a**) Whitson method; (**b**) Fast Newman method.

To compare how the benchmark methods organize the network under high-demand conditions, Figure 4 presents the partitioning process outputs of the Whitson and Fast Newman methods, and Figure 5 shows their corresponding final spatial partitioning results. The results indicate that the two methods follow different partitioning logics for coordinated control.

As shown in Figure 4a and Figure 5a, the Whitson method is primarily driven by correlation relationships between adjacent intersections. Strong correlations are mainly formed between neighboring nodes, leading to control subareas with clear proximity-based aggregation. This gives the partition boundaries relatively good local continuity and makes the result easy to interpret from the perspective of adjacent coordination demand. However, because the method mainly relies on local correlation strength, its ability to capture broader propagation relationships across the network remains limited. By contrast, Figure 4b and Figure 5b show that the Fast Newman method organizes the network through hierarchical community merging. Its final control subareas are more strongly shaped by topological cohesion and global community structure than by local traffic-state differences. This partitioning logic can produce structurally coherent subareas, but it does not explicitly account for how traffic disturbances propagate under dynamic traffic loading.

Overall, the key difference between the two benchmark methods lies in the basis on which subarea boundaries are formed. The Whitson method emphasizes local correlation and neighboring coordination, whereas the Fast Newman method emphasizes topological clustering and community structure. Both methods reveal useful structural characteristics of the network, but neither explicitly incorporates traffic-state propagation. Therefore, they provide meaningful benchmarks for evaluating whether the proposed method can generate propagation-consistent control subareas under dynamic traffic conditions.

### 4.5. Propagation Mechanism and Partitioning Result of the Proposed Method

**Figure 6:**
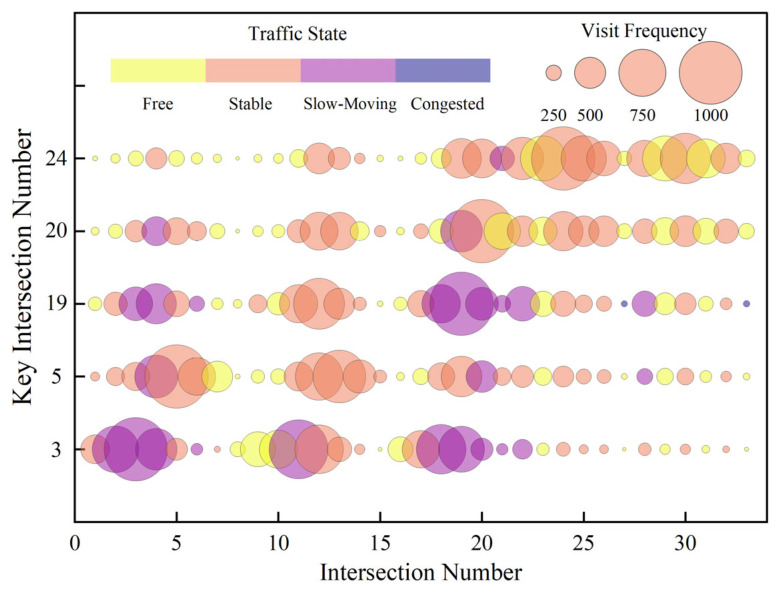
State-guided propagation patterns of representative seed intersections under the high-load condition.

**Figure 7:**
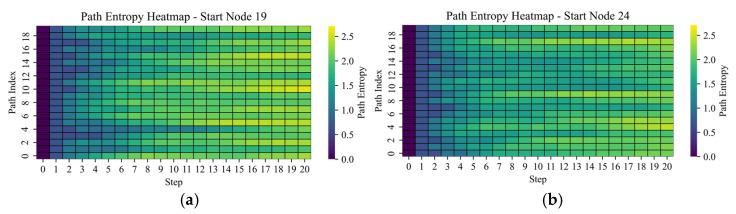
Representative path entropy evolution for selected seed intersections. (**a**) Start node 19; (**b**) Start node 24.

**Figure 8:**
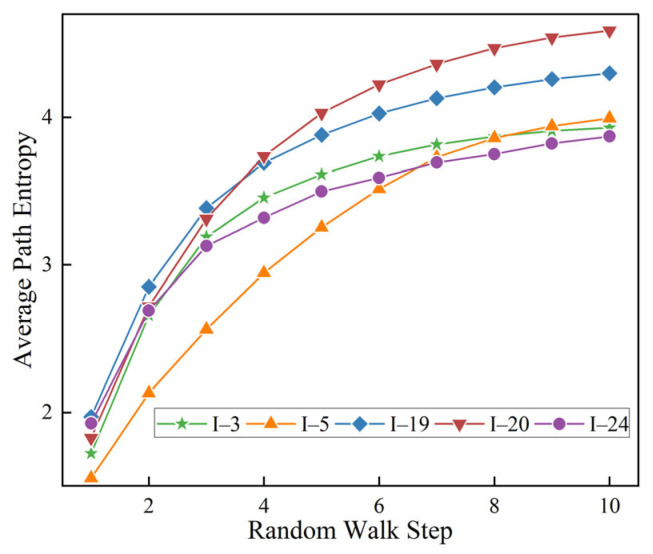
Convergence of average path entropy with increasing random walk steps.

**Figure 9:**
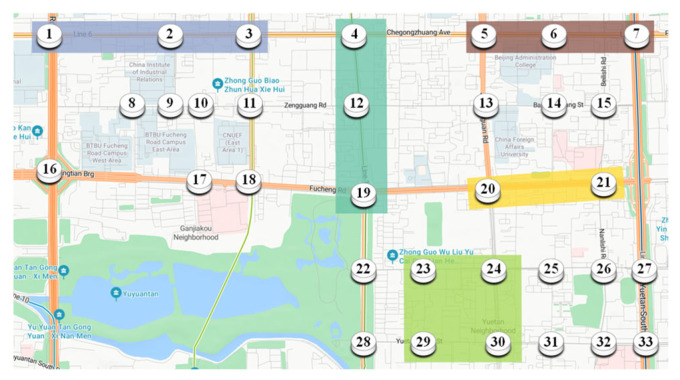
Control subarea partitioning result of the proposed method under the high-load condition.

To explain the partitioning mechanism of the proposed method, this section examines traffic-state propagation, path entropy evolution, and the resulting control subarea division. Unlike the two benchmark methods, which partition the network mainly according to local correlation or topological clustering, the proposed method identifies control subareas based on the consistency of traffic-state propagation. Figure 6 shows the state-guided propagation patterns, Figure 7 and Figure 8 show the evolution and convergence of path entropy, and Figure 9 presents the resulting control subarea partitioning result.

As shown in Figure 6, different seed intersections generate distinct propagation patterns. Bubble size represents node visit frequency during the random walk process, and node color represents the traffic-state category. For some seed intersections, high-frequency responses remain concentrated in nearby areas, whereas for others they extend along connected corridors or adjacent regions. In several representative cases, larger bubbles appear around slow-moving or congested nodes, suggesting that stronger propagation responses tend to emerge in more deteriorated parts of the network. This result indicates that the proposed method does not propagate traffic influence according to static adjacency alone, but adjusts the direction and range of propagation according to traffic-state labels.

The uncertainty of this propagation process is further illustrated in Figure 7 and Figure 8. In Figure 7, path entropy for the representative seed intersections increases rapidly during the first few random walk steps and then gradually stabilizes. Figure 8 shows the same trend at the aggregate level: the average path entropy rises sharply within the first few steps and becomes nearly stable after about 8–10 steps. Although the final entropy levels differ across intersections, the convergence pattern is consistent. This indicates that the proposed method can capture the main multipath diffusion characteristics of key intersections with a limited number of steps, while path entropy provides a stable representation of propagation uncertainty for subsequent control subarea division.

Based on these propagation and entropy characteristics, the resulting partitioning result is shown in Figure 9. The control subarea boundaries are shaped not only by local links or static topology, but also by the spatial organization of traffic-state propagation. Several nodes along the same corridor with continuous propagation relationships are grouped into the same control subarea, whereas some highly loaded intersections are not simply grouped into the same subarea but are organized with surrounding nodes that exhibit similar path response patterns. As a result, the proposed method helps avoid concentrating multiple severely congested intersections in a single control subarea and improves the internal consistency of propagation patterns within each subarea. The partitioning result in Figure 9 therefore reflects a propagation-consistent control subarea division rather than a purely structural clustering result.

### 4.6. Integrated Quantitative Evaluation of Network-Level Performance

**Figure 10:**
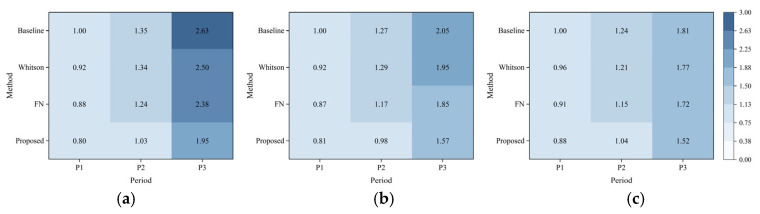
Stage-wise normalized direct traffic measures based on five simulation replications. (**a**) Average delay; (**b**) Average number of stops; (**c**) Average queue length.

This section evaluates the network-level control performance of different partitioning methods under dynamic traffic loading. Figure 10 first compares three direct traffic measures, namely average control delay, average number of stops, wand average queue length, in a stage-wise manner. The value in each heatmap cell is calculated from the corresponding network-level average for that period and then normalized by the corresponding baseline average in Period 1, with the reference value set to 1.00. Larger values thus indicate a heavier network operating burden.

As shown in Figure 10, all three direct traffic measures increase continuously from Period 1 to Period 3, indicating progressive deterioration as traffic demand rises. For average control delay, the baseline value increases from 1.00 in Period 1 to 2.63 in Period 3, whereas the proposed method remains at 1.95 in Period 3. The same pattern is observed for the average number of stops and average queue length: in Period 3, the baseline reaches 2.05 and 1.81, while the proposed method remains at 1.52 and 1.52, respectively. The Fast Newman method generally performs better than the Whitson method, but both remain visibly closer to the baseline in the higher loading periods. These results indicate that the proposed method is more effective throughout the loading process in restraining delay growth, reducing stop-and-go operations, and suppressing queue accumulation, and that its advantage becomes more pronounced under high-load conditions.

Table 4 summarizes the integrated performance of the three methods using the five aggregated metrics defined in Section 4.2. The values are reported as mean (range) over five independent VISSIM simulation replications. The proposed method ranks first on all five indicators, reaching mean values of 41.47%, 23.77%, 25.96%, 23.59%, and 15.08% for VRR, DRR, CMI, SRR, and QRR, respectively. The ranges across the five replications were limited and did not change the comparative ranking of the three methods, indicating that the performance advantage of the proposed method was not caused by a particular random seed.

Among the five metrics, VRR and CMI are particularly informative. The proposed method achieves a VRR of 41.47%, which is more than twice that of the Fast Newman method and nearly four times that of the Whitson method, indicating a much stronger reduction in the disparity of operating conditions across intersections. Its CMI reaches 25.96%, compared with 9.61% for the Fast Newman method and 5.29% for the Whitson method, showing that the proposed method is also more effective in relieving severe congestion at critical bottlenecks under the highest loading condition. These results are consistent with the mechanism analysis in Section 4.5, namely that control subareas determined according to traffic-state propagation consistency are better aligned with the actual spreading pattern of traffic influence.

The advantages of the proposed method in DRR, SRR, and QRR further indicate that its benefit is also reflected in several direct operational dimensions. Its DRR reaches 23.77%, compared with 9.71% and 4.27% for the Fast Newman and Whitson methods, showing a substantially larger reduction in network-level average delay. Its SRR reaches 23.59%, clearly exceeding the 10.10% of the Fast Newman method and the 4.34% of the Whitson method, indicating fewer repeated stops and smoother vehicle progression. Its QRR reaches 15.08%, compared with 6.08% and 1.90%, respectively, which indicates a stronger ability to suppress queue accumulation under dynamic traffic loading. Taken together, Figure 10 and Table 4 show that the proposed method not only maintains better direct operating conditions throughout the loading process, but also converts these improvements into stronger network-level coordinated control performance.

## 5. Conclusions and Future Work

This study investigated control subarea division for coordinated signal control under dynamic traffic loading. Existing methods based mainly on local correlation, traffic-state similarity, or topological cohesion may have limited ability to represent how traffic influence propagates across multiple paths in an urban road network. To address this limitation, this study developed a propagation-oriented control subarea division framework that integrates state-guided colored random walk and path entropy analysis. In this framework, intersection correlation degree and traffic-state labels are used to guide the random walk transition process, while path response distributions, path entropy, and KL-divergence-based distribution discrepancy are used to identify control subareas with internally consistent propagation patterns.

The VISSIM-based evaluation showed that the proposed method performed better than the Whitson and Fast Newman methods under dynamic loading conditions. The improvement was not limited to a single operational indicator, but was reflected in spatial delay balance, network-level delay reduction, bottleneck mitigation, stop reduction, and queue suppression. These results indicate that incorporating traffic-state propagation into the subarea division process can provide a more suitable basis for coordinated signal control than relying only on adjacent correlation or topological community structure.

The mechanism analysis further suggests that control subarea boundaries should be determined not only by whether intersections are spatially adjacent or structurally connected, but also by whether they exhibit similar path response patterns during traffic-state propagation. The state-guided colored random walk captures the direction and range of traffic influence diffusion, while path entropy provides an uncertainty-aware description of the propagation process. Therefore, the proposed method helps form control subareas that are more consistent with the internal propagation characteristics of the network, especially under high-demand conditions.

Several limitations remain. First, the validation was conducted on one representative urban road network, and the generalizability of the proposed method should be further examined using larger networks and more heterogeneous traffic scenarios. Second, the intersection correlation degree and traffic-state labels used in the random walk process were taken as model inputs; uncertainties in these inputs may affect the resulting path response distributions and subarea boundaries. Third, the evaluation was carried out in a simulation environment. Although VISSIM provides a controlled basis for comparing different partitioning schemes, additional empirical validation is still needed before field application.

Future work will focus on three aspects. First, larger-scale urban networks and more diverse demand patterns will be used to test the robustness of the proposed framework. Second, the traffic-state labels and intersection correlation degrees will be updated using richer and more dynamic traffic data, so that the subarea division process can better reflect time-varying network conditions. Third, the proposed partitioning framework will be further integrated with adaptive signal control strategies to explore online subarea updating and practical deployment under real-world traffic management conditions.

## Figures and Tables

**Table 1:** Parameter settings of the three partitioning methods.

Method	Parameters and Values
Whitson	Subarea partition threshold θ=0.35; $\mathrm{Average}\;\mathrm{travel}\;\mathrm{time}\;T_{W}$ $\mathrm{measured}\;\mathrm{by}\;\mathrm{Travel}\;\mathrm{Time}\;\mathrm{Sections};\;\mathrm{Directional}\;\mathrm{traffic}\;\mathrm{volume}\;q_{i}$ $\mathrm{and}\;\mathrm{maximum}\;\mathrm{traffic}\;\mathrm{volume}\;q_{\max}$ at upstream intersections obtained from Data Collection Points.
Fast Newman	Symmetric average correlation = False; merging direction determined by the direction yielding the maximum modularity increment.
Proposed	$\lambda_{1}=\lambda_{2}=0.15,$ α=0.3, β=0.1, step=10, $\;N_{walk}=1000$

**Table 2:** Sensitivity analysis of key parameters at the algorithmic level.

Parameter	Tested Values	Partition Agreement	Changed Intersections	Average KL Divergence	Average Path Entropy	No. of Subareas
$\lambda_{1}$	0.05–0.25	1.000–1.000	0	0.1486–0.1575	2.788–2.809	5
$\lambda_{2}$	0.05–0.25	0.968–1.000	0–1	0.1480–0.1614	2.784–2.811	5
α	0.1–0.5	1.000–1.000	0	0.1506–0.1542	2.797–2.801	5
β	0.05–0.20	1.000–1.000	0	0.1529–0.1530	2.798–2.799	5
step	4–15	0.892–1.000	0–7	0.0985–0.3401	2.360–2.976	5
$N_{walk}$	200–2000	0.939–1.000	0–12	0.1513–0.1599	2.795–2.799	5

**Table 3:** Robustness checks for baseline traffic deterioration.

Indicator	Classical ANOVA *p*	Welch ANOVA *p*	Kruskal–Wallis *p*	$\mathrm{Effect}\;\mathrm{Size}\;\eta^{2}$
Average queue length	0.048	0.098	0.177	0.061
Average stops	<0.001	<0.001	0.001	0.150
Average delay	<0.001	<0.001	<0.001	0.161
Average Vehicle count	<0.001	<0.001	0.003	0.151

**Table 4:** Integrated network performance metrics of different partitioning methods.

Method	VRR	DRR	CMI	SRR	QRR
Whitson	10.80 (10.21~11.19)	4.27 (4.08~4.43)	5.29 (4.94~5.56)	4.34 (4.09~4.70)	1.90 (1.81~2.04)
FN	18.51 (17.61~19.28)	9.71 (9.05~10.32)	9.61 (8.80~10.03)	10.10 (9.86~10.35)	6.08 (5.92~6.19)
Proposed	41.47 (38.91~44.54)	23.77 (22.97~24.57)	25.96 (25.34~27.08)	23.59 (22.65~24.05)	15.08 (14.22~15.77)

## Data Availability

Data are contained within the article.
